# Assessing Focused Antenatal Care Awareness and Utilization Among Pregnant Women in Enugu State, Nigeria: A Cross-Sectional Survey

**DOI:** 10.7759/cureus.38403

**Published:** 2023-05-01

**Authors:** Cherechi O Nwabueze, Chinyere C Okeke, Chimaobi O Nwevo, Lynda A Nwodo, Williams C Nwekpa, Peter I Nwaiwu

**Affiliations:** 1 Public Health, George Washington University, Washington, D. C., USA; 2 Medicine and Surgery, University of Nigeria Teaching Hospital, Enugu, NGA; 3 Community Medicine, University of Nigeria Teaching Hospital, Enugu, NGA; 4 Medicine and Surgery, University of Calabar Teaching Hospital, Calabar, NGA

**Keywords:** pregnancy care, antenatal clinic, antenatal care visits, womens health, south east nigeria, enugu, pregnant women, antenatal clinics, who, focused antenatal care

## Abstract

Introduction: Focused antenatal care (FANC) is a newer and better approach to antenatal care for pregnant women than the traditional model. FANC emphasizes individual assessment and decision-making by both the provider and the pregnant woman, resulting in better health outcomes for both mother and baby. Despite the adoption of FANC care in Nigeria, maternal mortality indices have not significantly decreased. This study aimed to assess the level of awareness and utilization of FANC among pregnant women in Nigeria, as well as the factors that influence its utilization.

Methods: This study was conducted in Enugu, Nigeria, using the antenatal clinics of three major tertiary hospitals. A cross-sectional design was used, and a sample size of 300 pregnant women was selected using systematic random sampling. Data were collected using a structured, self-administered questionnaire and analyzed using IBM Statistical Package for Social Sciences (SPSS) version 26. The findings were presented using frequencies, tables, charts, and figures, and Fisher's exact test was used to determine the relationship between respondents' knowledge of focused antenatal care and their demographic factors.

Results: A study involving 300 pregnant women in Nigeria found that only 15% of them had heard of focused antenatal care (FANC) and just 7.3% had good knowledge of its components, which was attributed to the low level of education among the respondents (X2=16.68, p=0.001). Health talks during antenatal visits were the most common source of information on FANC. The study also revealed that late initiation of antenatal care (n=144, 48%) in current pregnancy and (n=106, 54.6%) among those previously pregnant, as well as insufficient attendance, were identified as risk factors for maternal mortality. Long waiting times (n=196, 65.3%) and overcrowded healthcare facilities (n=110, 36.7%) were the major causes of dissatisfaction with antenatal care services among the respondents. Pregnant women preferred delivering at tertiary hospitals or private hospitals due to the perceived better quality of care and personal preference. These findings could inform targeted interventions to improve knowledge and awareness of FANC among pregnant women, particularly those with lower levels of education.

Conclusion: This study provides important insights into the low awareness and utilization of FANC among pregnant women in Enugu, Nigeria, highlighting the need for targeted interventions to improve knowledge and awareness of FANC. The study's findings have important implications for the development of maternal and child health policies and interventions aimed at improving the utilization of healthcare services during pregnancy and childbirth in Nigeria. Further research that includes qualitative methods could provide more nuanced information on pregnant women's experiences and perspectives on FANC.

## Introduction

Maternal mortality refers to deaths resulting from complications during pregnancy or childbirth. Unfortunately, maternal mortality rates remain unacceptably high worldwide [1,2]. In 2020, about 287,000 women died during and after pregnancy, resulting in an overall maternal mortality rate (MMR) of 223 per 100,000 live births. This translates to almost 800 maternal deaths daily, or roughly one maternal death every two minutes globally [1]. A woman’s lifetime risk of maternal death is the probability of a 15-year-old woman dying from a maternal cause; globally, this risk is one in 210 [1,2]. Almost 95% of these global maternal deaths occurred in low and lower-middle-income countries, most of which could have been prevented [1,2]. Of particular concern is Sub-Saharan Africa, which had a very high MMR of 545 per 100,000 live births, accounting for about 70% (202,000) of maternal deaths [1]. West Africa was the subregion in Sub-Saharan Africa with the highest MMR (754 per 100,000 live births) [1], with Nigeria being the most populous country in West Africa and having the highest number of maternal deaths (approximately 82,000), accounting for more than a quarter (28.5%) of all estimated global maternal deaths in 2020 [1,3].

Most maternal deaths are preventable or treatable with adequate antenatal care [2]. Antenatal care (ANC) refers to the medical procedures and care provided during pregnancy [4], including the clinical assessment of the pregnant woman and her fetus, aimed at achieving a favorable outcome for both the mother and child [5]. ANC services include detailed history taking, physical examination, laboratory work, and diagnostic investigations [5]. The traditional model of ANC, introduced in the United States in 1900 by social reformers and nurses [6], was based on the assumption that the more hospital visits a pregnant woman made, the better the outcome. It focused on the quantity or number of hospital visits and not on the quality of care [5]. The pregnant women were classified as low- or high-risk based on predetermined criteria [5]. They were expected to make up to 14 antenatal visits before delivery, regardless of their risk status, and only pregnancy-related issues were to be addressed at each visit [5]. This model proved ineffective in reducing maternal mortality due to difficulties in effective implementation, high resource consumption, and, most importantly, the challenge of predicting maternal outcomes because women classified as low-risk could develop complications, particularly during childbirth [5].

To address these challenges, the World Health Organization (WHO) introduced focused antenatal care (FANC) in 2002 [7]. FANC focuses on evidence-based interventions carried out at certain critical times during pregnancy, assuming that all pregnant women are at risk of developing complications and that quality, individualized care is more significant than the quantity of care [7]. It recommends a minimum of four visits to the pregnancy health care center for pregnancies without complications. The first visit should be before 12 weeks, or when a woman first thinks she is pregnant; the second visit should be around 26 weeks, or at least once in the second trimester; the third visit should be around 32 weeks of pregnancy; and the fourth visit between 36 and 38 weeks; thereafter, women are advised to return at 41 weeks or sooner if they experience danger signs [7]. This model of care aims to achieve early detection of complications and other potential complications, assists in the early identification and treatment of already established diseases, and makes pregnancy a family responsibility, with both the husband and the woman fully informed of the potential complications, birth preparedness, postnatal care, and planning for future child spacing and childbirth. The superiority of FANC to the traditional model of antenatal care in reducing maternal mortality has made it the recommended type of antenatal care [7].

Hence, in order to reduce maternal deaths through antenatal care, it is critical to link care with detecting and treating causes of maternal mortality by a skilled healthcare provider, which can only be achieved through the effective utilization of antenatal care services [8]. The high rate of maternal mortality in low-income countries reflects the inequalities in access to quality health services and highlights the gap between the rich and the poor [2]. Every woman deserves access to high-quality healthcare during pregnancy, childbirth, and the postpartum period. Unfortunately, women living in remote areas and those who are economically disadvantaged are least likely to receive adequate care. In Nigeria, the utilization rate of health services is alarmingly low, with significant disparities across states, geopolitical zones, and rural and urban areas, as well as differences based on education and social status [2]. Recent data indicates that only about 67% of pregnant women in Nigeria visited a skilled healthcare provider at least once during their pregnancy, compared to an average of 86% in other lower-middle-income countries [9,10]. The low utilization of antenatal care and institutional delivery in Nigeria has resulted in poor maternal outcomes [10], with a maternal mortality ratio of 814 per 100,000 live births and approximately 58,000 maternal deaths per year [11]. One in every 22 Nigerian women dies during pregnancy, childbirth, postpartum, or post-abortion, while the rate is only one in 4,900 in most developed countries [11]. Despite the simplicity and goal-oriented nature of focused antenatal care, several barriers hinder its complete adoption in Nigeria [12,13].

Although the severity of maternal mortality rates in Nigeria is apparent, there has been little progress in reducing these rates. This study aims to determine the adoption of focused antenatal care (FANC) at major tertiary hospitals in Enugu, Nigeria. Additionally, it aims to evaluate the level of awareness of FANC among pregnant women and the extent of utilization of available health services. Finally, identifying the major factors that influence the utilization of FANC is crucial and could provide guidance for future health interventions.

## Materials and methods

Study Area and Method

The study was carried out in Enugu, Nigeria, utilizing the antenatal clinics of three major tertiary hospitals that provide healthcare for a significant proportion of the population in that area. These hospitals were the University of Nigeria Teaching Hospital, Ituku Ozalla; the Enugu State University Teaching Hospital, Parklane; and the Mother of Christ Specialist Hospital, Ogui, Enugu. The target population consisted of all pregnant women attending these antenatal clinics, irrespective of their gestational age. The study received ethical approval from the Health Research and Ethics Committee at the University of Nigeria Teaching Hospital, Ituku Ozalla, Enugu (approval NHREC/05/01/2008B-FWA00002458-IRB00002323). Permission was also obtained from the other hospitals. Verbal and written informed consent was obtained from all participants, and measures were taken to ensure confidentiality and anonymity. Participants were informed that they could withdraw from the study at any time without any consequences. A cross-sectional study design was used for this study, and participants were selected based on daily clinic attendance registers, utilizing systematic random sampling to recruit every fourth pregnant woman after randomly selecting the first participant. The sample size of 300 participants was determined using a distinct population proportion formula based on the prevalence (22.4%) of the utilization of focused antenatal care in an urban area from a similar study [14]. A 5% significance level and error margin were used, and an additional 10% of the minimum sample size was added to account for potential non-response or other sources of data loss.

Data Collection and Analysis

Data collection was between January and June 2019, using a structured, self-administered questionnaire that was adapted from a previous study [15]. It was modified and pretested among 10 randomly selected pregnant women attending the antenatal clinic at Annunciation Specialist Hospital, Enugu, Nigeria, a tertiary hospital not selected for the study, to reduce bias and ensure that there were no ambiguous questions. For uneducated women who could not read in the English language, the questionnaire was translated into the local language (Igbo and Pidgin English) and subsequently re-translated before analysis. The Statistical Package for Social Science Software (SPSS) program, IBM Corp., version 26, was used to analyze the data, which was presented using frequencies, tables, charts, and figures. Fisher's exact test was used at a 5% level of significance to show the relationship between the respondents' knowledge of focused antenatal care and their demographic factors. A p-value of 0.05 was considered statistically significant.

## Results

This study enrolled a total of 300 pregnant women who received antenatal care at the three major tertiary hospitals. Half of the participants (50%) were between 20 and 29 years of age. The majority of the respondents identified as being of Igbo ethnicity (89.7%) and had completed secondary education (41%). Almost all of the participants resided in urban areas (95.7%), with the majority identifying as Christians (94.3%) and married (96%). Among the participants, petty trading (44%) was the most common occupation (Table 1). Most of the husbands of the respondents had attained a secondary level of education (48.7%), and over half (53.3%) were farmers. Only a small proportion, 0.7%, identified as professionals (Table 2).

**Table 1:** Socio-demographic characteristics of the pregnant women in the study

Variables	Values	Frequency (N=300)	Percentage (%)
Age	<20 years	14	4.7
	20-29 years	150	50.0
	30-39 years	123	41.0
	40-49 years	13	4.3
Tribe	Yoruba	19	6.3
	Igbo	269	89.7
	Hausa	10	3.3
	Others	2	0.7
Level of education	Primary	59	19.7
	Secondary	123	41.0
	Tertiary	109	36.3
	None	9	3.0
Religion	Christianity	283	94.3
	Islam	17	5.7
Marital status	Married	288	96.0
	Single	11	3.7
	Widowed	1	0.3
Occupation	Housewife	57	19.0
	Petty trader	132	44.0
	Student	12	4.0
	Farmer	5	1.7
	Professional	23	7.7
	Private sector	62	20.7
	Others	9	3.0
Residency	Urban	287	95.7
	Rural	13	4.3

**Table 2:** Socio-demographic characteristics of respondents’ husbands in the study

Variables	Values	Frequency (N=300)	Percentage (%)
Husband's level of education	Primary	37	12.3
	Secondary	146	48.7
	Tertiary	101	33.7
	None	16	5.3
Husband's occupation	Petty trader	77	25.7
	Student	6	2.0
	Farmer	160	53.3
	Professional	2	0.7
	Private sector	42	14.0
	Others	13	4.3

**Figure 1:**
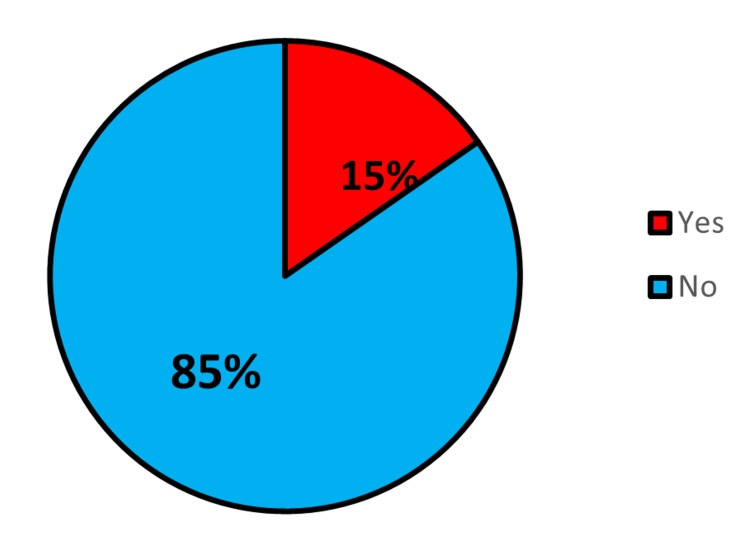
Level of awareness of focused antenatal care among the pregnant women in the study

**Figure 2:**
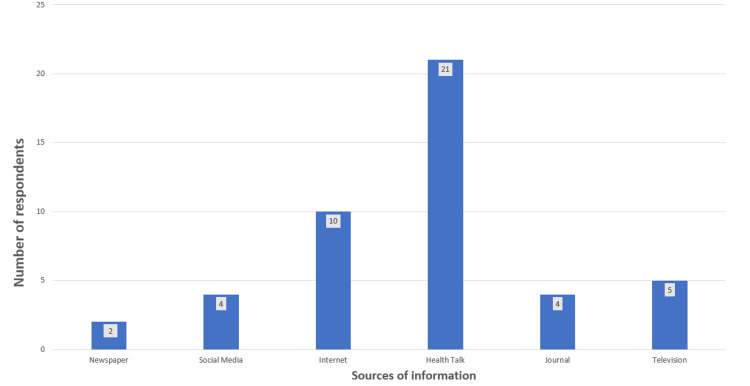
Sources of information on focused antenatal care among the respondents (46) that were aware of focused antenatal care

The study findings revealed that only about 15% (46) out of the 300 respondents reported being aware of focused antenatal care (Figure 1). Among those who were aware, 21 pregnant women reported that they received information on focused antenatal care through the health talks provided during antenatal visits, which was identified as the most common source of information on focused antenatal care compared to the other sources (Figure 2).

To obtain additional information on the participants' understanding of various aspects of focused antenatal care, including the WHO's standard recommendation, the study posed several questions. However, it should be noted that the respondents were not obligated to answer all questions. Only 62 of the participants attempted the first question, which asked for a definition of focused antenatal care. The findings showed that most of the respondents (84%) were unsure of the minimum number of visits recommended for focused antenatal care. Moreover, half of the respondents (50%) indicated that the optimal time to initiate antenatal care was between 16 and 20 weeks of gestational age (Table 3). In addition, the study found that the overall proportion of respondents with correct knowledge of focused antenatal care was 28.7% (Table 4).

**Table 3:** Respondents’ level of knowledge of focused antenatal care WHO: World Health Organization; FANC: focused antenatal care

Variables	Values	Frequency (N=300)	Percentage (%)
Definition of focused ANC	Skilled health care for pregnant women	6	2.0
	Professional healthcare for pregnant women to ensure the best health conditions for both mother and child	15	5.0
	A goal-oriented antenatal care approach to delivering evidence-based interventions carried out at four critical times during pregnancy	22	7.3
	Antenatal care emphasizes individualized assessment and decision-making that involves both the healthcare provider and the pregnant woman	19	6.3
	Antenatal care that emphasizes targeted and individualized care planning and birth planning	5	1.7
Minimum number of visits for focused ANC	1	2	0.7
	2	5	1.7
	3	6	2.0
	4	26	8.7
	5	9	3.0
	Not sure	252	84.0
Expected gestational age to book for ANC	4 to 8 weeks	14	4.7
	8 to 12 weeks	38	12.7
	12 to 16 weeks	65	21.7
	16 to 20 weeks	150	50.0
	20 to 24 weeks	7	2.3
	32 to 36 weeks	1	0.3
	Not sure	25	8.3

**Table 4:** Proportion of respondents with correct knowledge of focused antenatal care ANC: antenatal care; WHO: World Health Organization

Variable	Frequency (N =300)	Percentage (%)
Focused ANC care is a goal-oriented antenatal care approach to delivering evidence-based interventions carried out at four critical times during pregnancy	22	7.3
The minimum number of visits for focused ANC is four visits	26	8.7
The expected gestational age to book for focused ANC is 8 to 12 weeks	38	12.7

**Figure 3:**
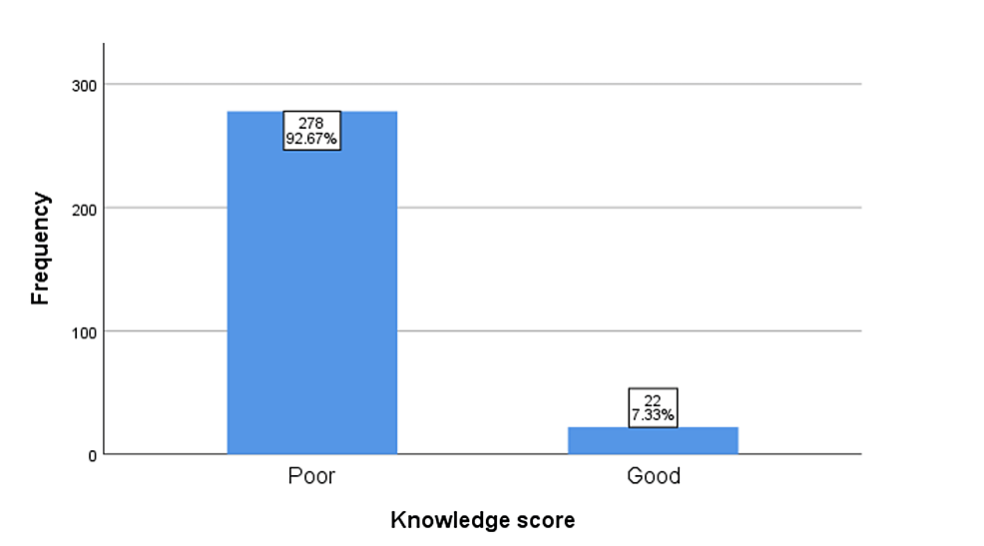
A bar chart showing the knowledge score of all respondents Knowledge score: n%

To quantify the level of knowledge of focused antenatal care among the respondents, a score of '1' was assigned to correct responses to questions (Table 4), and a total score of 3 was possible. Respondents with a score of 0-1 were classified as having "poor knowledge", while those with a score of "2-3" were classified as having "good knowledge". The findings showed that the majority of the respondents (92.67%) had "poor knowledge" of focused antenatal care, while only a small proportion (7.33%) had "good knowledge" (Figure 3). Furthermore, Fisher's exact test was conducted to examine the statistical relationship between the participants' demographic factors and their knowledge of focused antenatal care. The findings indicated a significant association between knowledge of FANC and level of education (X2 = 16.678, p-value = 0.001). A considerably higher proportion (77.3%) of participants with 'good knowledge' had tertiary education (Table 5).

**Table 5:** Relationship between the demographic factors and the respondents’ knowledge of focused antenatal care X2 = Fisher’s exact test; *Test statistic is significant at 0.05 level; ANC: antenatal care

Variable		Knowledge score (%)			
		Poor knowledge	Good knowledge	X^2^	p-Value
Age (in years)	< 20	14 (5.0)	0 (0.0)	2.630	0.359
	20 - 29	135 (48.6)	15 (68.2)		
	30 - 39	116 (41.7)	7 (31.8)		
	40 - 49	13 (4.7)	0 (0.0)		
Level of education	Primary	59 (21.2)	0 (0.0)	16.678	0.001*
	Secondary	118 (42.4)	5 (22.7)		
	Tertiary	92 (33.1)	17 (77.3)		
	No formal education	9 (3.2)	0 (0.0)		

In the study, about 48% of respondents booked for ANC between 16 and 20 weeks of gestational age. A majority of the respondents (89.7%) utilized services at tertiary hospitals for all their antenatal visits, while some also used other healthcare facilities. The utilization of the services rendered during antenatal care was over 50% for most services, except for the prevention of mother-to-child HIV transmission, which had a utilization rate of 16%. About 50% of the women did not have a prior appointment for their visit but were at the hospital due to a new complaint (Table 6).

**Table 6:** Level of the utilization of focused antenatal care among all respondents in the study ANC: antenatal care; IPT: intermittent preventive treatment; VDRL: venereal disease research laboratory test; HBsAg: hepatitis B surface antigen; HCV: hepatitis C virus

Variables	Values	Frequency (N=300)	Percentage (%)
Gestational age for ANC booking	4 to 8 weeks	14	4.7
	8 to 12 weeks	42	14.0
	12 to 16 weeks	54	18.0
	16 to 20 weeks	144	48.0
	20 to 24 weeks	10	3.3
	24 to 28 weeks	1	0.3
	Can’t remember	35	11.7
Health facility	Primary	2	0.7
	Secondary	12	4.0
	Tertiary	269	89.7
	Private	17	5.7
Services rendered during ANC visits	Serologic screening for syphilis	205	68.3
	Anti-tetanus immunization	181	60.3
	Prevention of mother-to-child HIV transmission	48	16.0
	IPT for malaria	235	78.3
	VDRL screening	168	56.0
	HBsAg and HCV screening	163	54.3
Reason for current ANC visit	On schedule	111	37.0
	A new complaint that needs to be managed by the doctor	150	50.0

Concerning the prospective plan for the current pregnancy, the majority of the respondents (76.3%) indicated a preference for delivery at a tertiary hospital, followed by 14.3% who preferred private hospitals. A small percentage expressed a preference for primary hospitals (1.0%) or traditional birth attendants (1.3%). Self-preference was the primary reason for the choice of delivery center (29.3%), followed by proximity (27.7%) (Table 7).

**Table 7:** The prospective plan of the respondents for the current pregnancy

Variable	Value	Frequency (N=300)	Percentage (%)
Preference for delivery	Traditional birth attendant	4	1.3
	Prayer home	0	0.0
	Private	43	14.3
	Primary	3	1.0
	Secondary	21	7.0
	Tertiary	229	76.3
Reason for preference	Cheaper	49	16.3
	Proximity	83	27.7
	Self-preference	88	29.3
	Attitude and cooperation of the health workers	71	23.7
	Lack of transportation	1	0.3
	Others	1	0.3

Out of the 300 respondents, 139 (46.3%) reported that the distance from home to the healthcare facility was 30 minutes. The majority of the respondents (42.7%) used commercial vehicles as their means of transportation, while 105 (35%) used commercial tricycles. Almost all respondents (89.3%) reported that they have never felt reluctant to attend the scheduled ANC visit. Among the few who did, 21 (7%) cited illness as the major cause of discouragement. The waiting time at ANC visits was reported as one to two hours by 167 (55.7%) respondents, and long waiting hours were the major cause of dissatisfaction in the ANC facilities, as reported by 65.3% of the respondents (Table 8).

**Table 8:** Factors affecting the utilization of focused antenatal care ANC: antenatal care

Variable	Values	Frequency (N=300)	Percentage (%)
Distance from home to health facility	30 minutes	139	46.3
	1 hour	134	44.7
	1 to 2 hours	24	8.0
	2 to 3 hours	3	1.0
	3 to 4 hours	0	0.0
	More than 4 hours	0	0.0
Means of transport	Tracking	4	1.3
	Commercial motorcycle	4	1.3
	Personal motorcycle	3	1.0
	Commercial tricycle	105	35.0
	Personal car	40	13.3
	Commercial vehicle	128	42.7
	Others	3	1.0
Ever felt reluctant to attend a scheduled visit	Yes	32	10.7
	No	268	89.3
Causes of discouragement from visiting the antenatal clinic	Not feeling ill	21	7.0
	No money for transport	1	0.3
	Tired and exhausted from work	10	3.3
	Far distance to the facility	7	2.3
	No money for maternal health services	7	2.3
	Others	3	1.0
Waiting time at the ANC clinic	Less than 30 minutes	20	6.7
	30 to 60 minutes	18	6.0
	1 to 2 hours	167	55.7
	More than 2 hours	80	26.7
	Not sure	15	5.0
Causes of dissatisfaction at the ANC facility	Long waiting hours (> 1 hour)	196	65.3
	Unavailability of maternal health services	8	2.7
	Movement up and down the facility	35	11.7
	Overcrowded health facility	110	36.7
	Others	6	2.0

About 194 of the respondents reported having had a previous pregnancy. Of those, 54.6% booked antenatal care between 16 and 20 weeks of gestation, while approximately 25.2% booked by at least 12 weeks. Additionally, around 38.7% had attended up to six antenatal care visits during their last pregnancy (Table 9).

**Table 9:** The level of utilization of antenatal care (ANC) during respondents' previous pregnancy

Variable		Frequency (N=300)	Percentage (%)
Respondents who have had a previous pregnancy		194	64.7
Variable	Value	Frequency (N=194)	Percentage (%)
Gestational age at the time of booking antenatal care during the previous pregnancy	4 to 8 weeks	4	2.1
	8 to 12 weeks	45	23.1
	12 to 16 weeks	34	17.5
	16 to 20 weeks	106	54.6
	20 to 24 weeks	2	1.0
	24 to 28 weeks	1	0.5
	28 to 32 weeks	1	0.5
	32 to 36 weeks	1	0.5
Number of antenatal care visits attended during last pregnancy	1	4	2.1
	2	7	3.6
	3	17	8.8
	4	57	29.4
	5	34	17.5
	6	75	38.7

## Discussion

Nigeria, which is the largest economy in Africa and the sixth most populous country in the world, comprises approximately 2.8% of the total global population [3,16,17]. Despite having vast human and natural resources, Nigeria faces significant developmental challenges, including poor reproductive health indices and high maternal mortality ratios [12,16]. The nationally adopted antenatal care model in Nigeria is focused on antenatal care, as evidenced by the training and orientation package of the Nigerian Federal Ministry of Health and malaria action [18]. This study aimed to assess the level of awareness and utilization of FANC among pregnant women and the factors that influence its utilization.

The study involved 300 respondents and showed a very low level of awareness of FANC among pregnant women. Only 15% of them had ever heard of FANC, and an even smaller proportion (7.3%) had good knowledge of its major components. This can be attributed to the low level of education among the respondents (X2=16.68, p=0.001). Although secondary education was the most common highest level of education (n=123, 41%), a significantly higher proportion (n=17, 77.3%) of respondents with good knowledge of FANC had tertiary education. A similar study conducted in Ido Ekiti, Nigeria, where 50.2% of the respondents had a tertiary level of education, reported an awareness level of FANC of 95% [19]. This suggests that higher education can increase women’s awareness and knowledge of ANC service utilization and its consequences, enabling them to make informed healthcare decisions. Additionally, higher education is linked to increased knowledge of obstetric complications, which can result in improved utilization of ANC services [20]. This study also found that health talks during antenatal visits were the most common source of information on FANC. This finding could be useful in developing targeted interventions to improve knowledge and awareness of FANC among pregnant women, particularly those with lower levels of education. This could include providing health education materials and training for healthcare providers to ensure that accurate and up-to-date information is provided during antenatal visits.

The World Health Organization (WHO) recommends initiating antenatal care in the first trimester and having at least eight antenatal visits during pregnancy [21]. However, many women in developing countries, including Nigeria, do not adhere to this recommendation [22]. Research has consistently demonstrated an association between the timing of antenatal care initiation and health outcomes for mothers and babies [23]. The study revealed that a large proportion of the respondents initiated antenatal care between 16 and 20 weeks of gestational age, both in their current pregnancy (n=144, 48%) and among those who had been previously pregnant (n=106, 54.6%). However, many pregnant women have misconceptions about the purpose of antenatal care and may view it solely as a curative service rather than a preventive one. This can impede the proper utilization of focused antenatal care services [24]. Late initiation of antenatal care and insufficient attendance are serious issues of concern in Nigeria and have been identified as risk factors for maternal mortality [18,24]. Health education is therefore needed to improve utilization.

Long waiting times (n=196, 65.3%) and overcrowded healthcare facilities (n=110, 36.7%) were the major causes of dissatisfaction with antenatal care services among respondents. This highlights the importance of addressing infrastructure and service delivery issues in antenatal care facilities to ensure that pregnant women receive timely and quality care. Most of the respondents indicated a preference for delivering at a tertiary hospital (n=229, 76.3%) or private hospital (n=43, 14.3%) due to the perceived better quality of care and availability of advanced medical facilities. Self-preference was the most common reason cited (n=88, 29.3%), indicating that the respondents prioritize their personal preferences when making healthcare decisions. Proximity to the healthcare facility was also an important factor (n=83, 27.7%), suggesting that access to healthcare facilities plays a crucial role in the decision-making process. It is important to note that the study only assessed the prospective plans of respondents for their current pregnancy and may not reflect their actual utilization of healthcare services during delivery. Future studies could examine the factors that influence the actual utilization of healthcare services during delivery.

Limitations

The study has some limitations that should be considered when interpreting the findings. Firstly, the study only included pregnant women who attend public hospitals, thus limiting the generalizability of the findings to those who seek care in private hospitals or use traditional birth attendants. Secondly, the cross-sectional design of the study only provides a snapshot in time and does not establish causality. Furthermore, self-reported data may be subject to desirability, measurement, and recall bias, particularly among multiparous women who may not accurately remember events from their past pregnancies. Finally, more qualitative research, such as in-depth interviews or focus group discussions with pregnant women, may provide a more nuanced and detailed understanding of the factors that influence the utilization of FANC. Despite these limitations, this study highlights the need for targeted interventions to improve awareness and utilization of focused antenatal care among pregnant women in Nigeria.

## Conclusions

This study highlights the low level of awareness and knowledge of FANC among pregnant women in Nigeria and the need for targeted interventions to improve awareness and utilization of FANC. Education was found to be a significant predictor of knowledge of FANC, with higher levels of education associated with better knowledge. Health talks during antenatal visits were identified as the most common source of information on FANC. The study also identified late initiation and insufficient attendance of antenatal care, as well as infrastructure and service delivery issues, as major concerns. Improving accessibility to healthcare facilities and promoting patient-centered care are crucial to improving the utilization of antenatal and delivery services. Long waiting times and overcrowded health facilities were identified as the major causes of dissatisfaction with antenatal care services among respondents.

This study emphasizes the need for targeted interventions to improve awareness and utilization of focused antenatal care among pregnant women in Nigeria, particularly those with lower levels of education. Healthcare providers should be trained to provide accurate and up-to-date information on FANC during antenatal visits. Addressing infrastructure and service delivery issues in antenatal care facilities is necessary to ensure that pregnant women receive timely and quality care. Efforts to improve access to healthcare facilities, reduce waiting times, and improve the quality of care in both public and private healthcare facilities can help increase the uptake of antenatal care services. Overall, the study provides important insights for policymakers and healthcare providers to improve maternal and child health outcomes in Nigeria.

The study has some limitations that should be considered when interpreting the findings. Firstly, the study only included pregnant women who attend public hospitals, thus limiting the generalizability of the findings to those who seek care in private hospitals or use traditional birth attendants. Secondly, the cross-sectional design of the study only provides a snapshot in time and does not establish causality. Furthermore, self-reported data may be subject to desirability, measurement, and recall bias, particularly among multiparous women who may not accurately remember events from their past pregnancies. Finally, more qualitative research, such as in-depth interviews or focus group discussions with pregnant women, may provide a more nuanced and detailed understanding of the factors that influence the utilization of FANC. Despite these limitations, this study highlights the need for targeted interventions to improve awareness and utilization of focused antenatal care among pregnant women in Nigeria.
